# Endoplasmic reticulum stress in pancreatic β-cell dysfunctionality and diabetes mellitus: a promising target for generation of functional hPSC-derived β-cells *in vitro*

**DOI:** 10.3389/fendo.2024.1386471

**Published:** 2024-06-20

**Authors:** Abdoulaye Diane, Asma Allouch, Razik Bin Abdul Mu-U-Min, Heba Hussain Al-Siddiqi

**Affiliations:** Diabetes Research Center, Qatar Biomedical Research Institute (QBRI), Hamad Bin Khalifa University (HBKU), Qatar Foundation (QF), Doha, Qatar

**Keywords:** β-cells, endoplasmic reticulum, stress, insulin, diabetes mellitus, hPSC-derived β-cells

## Abstract

Diabetes mellitus (DM), is a chronic disorder characterized by impaired glucose homeostasis that results from the loss or dysfunction of pancreatic β-cells leading to type 1 diabetes (T1DM) and type 2 diabetes (T2DM), respectively. Pancreatic β-cells rely to a great degree on their endoplasmic reticulum (ER) to overcome the increased secretary need for insulin biosynthesis and secretion in response to nutrient demand to maintain glucose homeostasis in the body. As a result, β-cells are potentially under ER stress following nutrient levels rise in the circulation for a proper pro-insulin folding mediated by the unfolded protein response (UPR), underscoring the importance of this process to maintain ER homeostasis for normal β-cell function. However, excessive or prolonged increased influx of nascent proinsulin into the ER lumen can exceed the ER capacity leading to pancreatic β-cells ER stress and subsequently to β-cell dysfunction. In mammalian cells, such as β-cells, the ER stress response is primarily regulated by three canonical ER-resident transmembrane proteins: ATF6, IRE1, and PERK/PEK. Each of these proteins generates a transcription factor (ATF4, XBP1s, and ATF6, respectively), which in turn activates the transcription of ER stress-inducible genes. An increasing number of evidence suggests that unresolved or dysregulated ER stress signaling pathways play a pivotal role in β-cell failure leading to insulin secretion defect and diabetes. In this article we first highlight and summarize recent insights on the role of ER stress and its associated signaling mechanisms on β-cell function and diabetes and second how the ER stress pathways could be targeted *in vitro* during direct differentiation protocols for generation of hPSC-derived pancreatic β-cells to faithfully phenocopy all features of bona fide human β-cells for diabetes therapy or drug screening.

## 1 Introduction

Diabetes mellitus (DM) is a chronic and complex metabolic disorder that results from a defect in insulin secretion, action, or both (1). The recent report of the international diabetes federation (IDF) estimated that 537 million adults (20–79 years) worldwide are living with diabetes (www.idf.org). The two main forms of DM are Type 1 and Type 2 diabetes (American Diabetes Association 1997 Report). Type 1 diabetes (T1DM) accounting ~10% of diabetic patients, is characterized by a selective autoimmune destruction of pancreatic β-cells leading to nearly complete loss of insulin production that typically develops over several years. Type 2 diabetes (T2DM), the most common affecting > 90% of people diagnosed with DM, results from an inability of pancreatic β-cells to produce sufficient insulin to stimulate glucose utilization by peripheral metabolically active organs to maintain glucose homoeostasis (2). Now, it is well acknowledged that both T1DM and T2DM converge on impaired insulin secretion and uncontrolled hyperglycemia secondary to pancreatic β-cell dysfunctionality, ultimately necessitating insulin therapy. While exogenous insulin administration option is considered as a life-saving treatment, it is unfortunately associated with acute episodes of hypoglycemia and weight gain in significant number of patients (3). Transplantation of islets isolated from deceased donors or surrogate insulin-producing β-cells from human pluripotent stem cells are an effective alternative approach to restore normoglycemia when endogenous β-cells have already been practically depleted (3–5). Human pluripotent stem cells (hPSC), including induced pluripotent stem cells (iPSC) and embryonic stem cells (ESC) can be differentiated virtually to any cell type in the body. Also due to their infinite self-renewal competency, they are a good alternative source to cadaveric islets. More strikingly, stem cell-derived pancreatic pseudoislets generated *in vitro* could potentially become an infinite source of insulin-secreting β-cells for a potential diabetes therapy. Over the last two and half decades, many efforts have been made on developing and implanting *in vitro* protocols to successfully differentiate hPSCs into insulin-producing β-cells with key features of bona fide mature β-like cells using multi-stages directed differentiation protocols that recapitulate and phenocopy all specific stages of pancreas embryogenesis (4, 6–8).

However, many protocols developed to differentiate stem cells into insulin-expressing β-cells *in vitro* have faced a roadblock: the resulting β-cells often exhibit an immature phenotype with impaired glucose-stimulated insulin secretion (GSIS) compared to cadaveric islets. This limitation highlights the need for further optimization of differentiation protocols to achieve the generation of fully mature and functional hPSC-derived β-cells. Multiple mechanisms underlie defective insulin secretion associated with β-cell dysfunction (9). Notably, accumulating evidence implicates endoplasmic reticulum (ER) stress and dysregulated ER stress signaling in β-cell failure, potentially contributing to insulin secretion defects and diabetes development (10–12). ER is an organelle that executes vital biological roles in the organism. It serves as a cellular hub for protein biogenesis, orchestrating posttranslational modifications, protein folding and assembly, and acting as a critical reservoir for calcium (Ca^2+^) storage (13, 14). It has been reported in both human studies and animal models that under condition of chronic metabolic disorders, ER stress is activated in several key metabolically actives tissues, including the liver, muscle, adipose, and pancreas (15). β-cell loss is a pathological component of both T1DM and T2DM, with recent reports indicating that ER stress plays a role in this process (16). Indeed, several reports have suggested that ER dysfunction exacerbates DM (17–19). The specialized function of β-cells, involving the constant synthesis and release of insulin in response to nutrient and hormone stimulation, makes them particularly susceptible to ER stress (11, 20, 21). Furthermore, in normal and healthy condition, over 50% of the total mRNA present in β-cell is allocated to insulin synthesis. Moreover, unfolded protein response (UPR), which is activated in response to ER stress aimed at restoring ER homeostasis, plays a vital for the maintenance of the integrity and function of β-cells. Therefore, maintaining proper ER proteostasis is critical for a normal function of β-cells. In this review, we will document and discuss the current understanding of the role of ER stress and its associated signaling mechanism on β-cell function and diabetes and how ER stress could be targeted *in vivo* for therapeutic opportunities and *in vitro* during direct differentiation protocols to generate totally functional hPSC-derived pancreatic β-cells for diabetes therapy and drug screening.

## 2 Endoplasmic reticulum stress or ER stress

**Figure 1:**
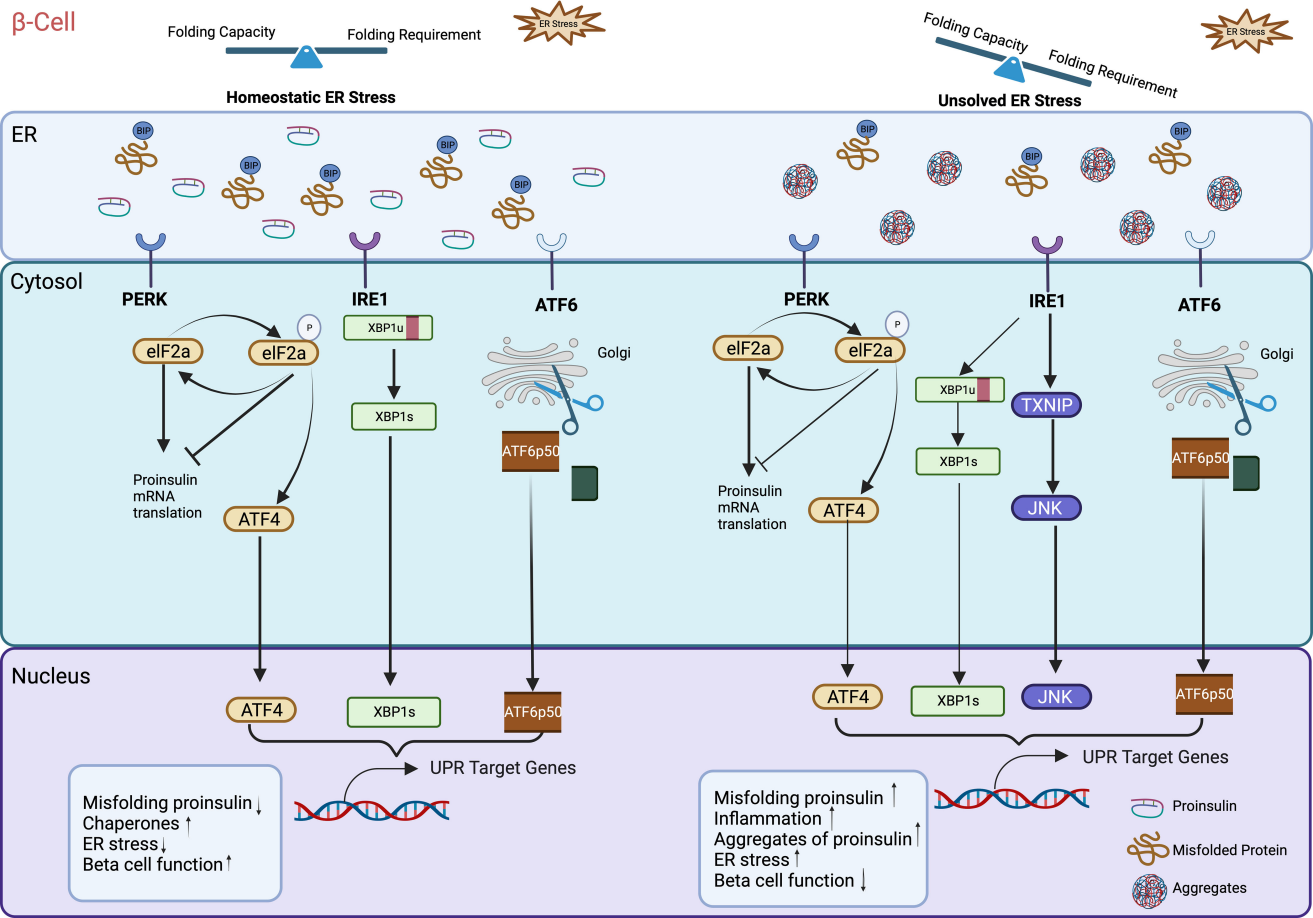
Unfolded protein response (UPR) dysfunction in β-cell ER. Increased proinsulin synthesis in response to nutrient demand directly contributes to physiological ER stress in pancreatic β-cells. In the absence of stress, the three UPR sensors IRE1, ATF6, and PERK stay inactive monomers bound to the ER chaperone BiP. Under normal healthy ER stress conditions when misfolded proinsulin accumulation exceeds the ER’ clearance capacity, BiP detaches from the UPR transmembrane sensors and binds to the exposed hydrophobic domains of the misfolded proinsulin, thus freeing PERK, ATF6, and IRE1. This triggers the activation of downstream signaling of these three UPR sensors and subsequently UPR target genes that help to maintain ER homeostasis by increasing molecular chaperones, reducing misfolded proinsulin retention. However, excessive or prolonged increased influx of newly synthesized proinsulin into the ER can exceed the ER capacity (folding requirement outstrips folding capacity), triggering the production of misfolded proinsulin aggregates. This activates and upregulates UPR target genes to induce inflammation, and β-cell failure. Illustration created with Biorender.com.

The endoplasmic reticulum (ER) was first discovered by Emilio Veratti in the late 19th century as Sarcoplasmic Reticulum in muscle fibers. The use of electron microscopy allowed Keith Porter in the 1940s to first visualize the morphology of this new organelle and named it as “endoplasmic reticulum” (22). The ER is the largest single structure present in most eukaryotic cell types (23) and consists of a range of interconnected shapes, including sheets, tubules, and lumen. While the ER lumen’s physical separation from the cytoplasm ensures distinct functional domains, its continuous connectivity with the nuclear membrane facilitates nuclear-cytoplasmic communication (24). Based on morphological structures, the ER can be divided into two distinct forms: the rough endoplasmic reticulum (RER) and the smooth endoplasmic reticulum (SER). This morphological difference is associated with distinct functional roles: SER is primarily responsible for the synthesis of phospholipids and cholesterol, while the central role of RER is the synthesis and export of proteins and glycoproteins. Overall, the ER is essential organelle and acts as a central player in the synthesis, modification, quality control, protein trafficking, and sterol/lipid synthesis (25). It also serves as a key site for mobilization and regulation of the Ca^2+^ release (26). Acting as a major synthetic organelle, the ER’s critical role in protein synthesis renders it extremely and highly sensitive to perturbations in homeostasis. Following synthesis and before leaving the ER, all newly synthesized proteins undergo meticulous quality control, involving protein folding, assembly, and post-translational modifications and only properly folded proteins are transported to the Golgi. Therefore, when misfolded or unfolded proteins accumulate within the ER, a cellular stress response named the UPR is triggered. UPR is a well conserved, intracellular signaling pathway in eukaryotic organisms. In yeast, more than 300 genes involved in all aspects of ER function, including protein folding are activated by the UPR. ER stress manifests when the functional demand of the organelle exceeds its protein folding capacity which subsequently leads to the accumulation of unfolded or misfolded proteins in the ER lumen. To restore ER homeostasis, cells activate the ER stress response that helps to limit the amount of newly synthesized proteins and increase the production of chaperones, specialized molecules that assist to fold unfolded proteins. In mammalian cells, such as β-cells, it is well documented that the UPR is classically controlled by three canonical ER-resident transmembrane proteins to respond to stress: ATF6 (activating transcription factor 6); IRE1 (inositol requiring 1); and PERK/PEK (PKR-like endoplasmic reticulum kinase/pancreatic eIF2a kinase), which each produce a transcription factor (ATF4, XBP1s, ATF6, respectively), resulting in activation of transcription of ER stress-inducible genes (**Figure 1**). Each of these proteins employ distinct mechanisms and signaling pathways to regulate the production of proteins crucial for ER function. This allows cells to dynamically adjust the protein folding capacity of the ER to match the demand. Mammalian cells have three canonical ER-resident transmembrane sensor pathways working in parallel, while only IRE1 pathway exists in yeast. To face this challenge, cells, particularly secretory cells, meticulously monitor the state of the ER protein folding. This control relies heavily on diverse chaperones like the immunoglobulin heavy chain binding protein, BiP (also called GRP78) belonging to the heat shock protein (HSP) families (27–30). Under normal and unstressed conditions, the concentration of BiP in the ER lumen exceeds the concentration of unfolded proteins (e.g. proinsulin), so free BiP constitutively binds to the three ER transmembrane sensors (ATF6, IRE1 and PERK), preventing their activation (**Figure 1**). However, when misfolded proteins (e.g.proinsulin) accumulate in the ER, BiP detaches from the UPR transmembrane sensors and binds to the exposed hydrophobic domains of the unfolded proteins (in this particular context, proinsulin) thus freeing PERK, ATF6, and IRE1. This results in the activation of specific downstream signaling pathways associated with the three UPR effector proteins—ATF6, IRE1, and PERK (see **Figure 1**) (31). These pathways manage global protein synthesis and chaperone expression, thereby promoting ER homeostasis.

## 3 Adaptive UPR to preserve β-cell function

The ER is a very important intracellular organelle where newly synthesized proteins, including proinsulin undergo folding into their unique three-dimensional (3D) conformations that are required for release into the extracellular space. Therefore, maintaining ER homeostasis is crucial for ensuring proper protein folding, assembly, and secretion (32). Notably, a balanced cellular environment within the ER is vital for various secretory cells such as pancreatic β-cells that are more susceptible to ER stress than other cells due to the high rate of proinsulin synthesis turnover. Pancreatic β-cells play an essential role in maintaining glucose homeostasis. They ‘sense’ changes in plasma glucose levels and other secretagogues such as neurotransmitters and circulating hormones by synthesizing and releasing insulin accordingly. In human, the high insulin synthesis turnover in response to increased secretory demand—inherent to the amounts of ingested food/meal—poses a great challenge on the protein folding machinery within the β-cell ER. To face this challenge, β-cells activate the UPR accordingly for a proper proinsulin folding inside the ER. This constitutes a regulated process vital for β-cell function and survival. Gain-and loss-of-function studies in both animal and human with specific mutations have established an essential role of the adaptive UPR transducers in pancreatic β-cell function. For example, studies investigating the effects of Perk deletion in mice, revealed that both young adult and mature adult mice resulted in hyperglycemia associated with loss of islet and β-cell architecture (33), and its mutation in humans causes β-cell failure associated with neonatal diabetes mellitus (34), highlighting its role in maintaining islet function. ATF6, a second UPR sensor, is also important for β-cell function as ATF6alpha-null mice on high-fat diet exhibit glucose intolerance due to pancreatic β-cell failure (35). Furthermore, genetic variations in ATF6 are associated with prediabetes in individuals of Chinese Han descent (36) and with Type 2 diabetes in Pima Indians (37). Additionally, IRE1 pathway, the third UPR transducer is also reported to be essential for glucose-stimulated insulin secretion and protection of β-cells. Mice lacking IRE1 develop DM due to proinsulin synthesis defect (38). Collectively, these findings indicated that adaptive UPR is required for normal β-cell function.

## 4 ER stress in β-cell dysfunction and diabetes

Pancreatic β-cells are highly sensitive to excessive ER stress and any defect in β-cells’ ability to maintain ER homeostasis can lead to their failure and apoptosis and the consequent development of DM (39, 40). It is now well-established that the UPR is important for β-cell function (41) and substantial evidence has demonstrated that both type 1 and type 2 polygenic diabetes, although caused by different mechanisms, share a common features: the presence of ER stress in β-cells (42).

T1DM and T2DM are the two major types of polygenic DM characterized by distinct underlying mechanisms. Relevant studies have now well demonstrated that these two forms of diabetes commonly share an enhanced ER stress in pancreatic β-cells that negatively impacts insulin secretion (21, 43, 44). The induction of β-cell ER stress by proinflammatory cytokines such as IL-1β and IFN-γ (45) implies that ER stress might contribute to pancreatic β-cell loss in T1DM. These cytokines activate the ER transmembrane sensor pathway and hamper β-cell defenses by inhibiting ER chaperones (43, 45). Thus, islet sections from T1DM patients showed increased levels of activating transcription factor 3 (ATF3), C/EBP homologous protein (CHOP) and BiP (46, 47).

T2DM is the more common type of polygenic DM, characterized by insulin resistance together with metabolic stress in metabolically relevant organs including pancreas. Increased β-cell workload due to sustained insulin resistance can trigger persistent ER stress and ultimately leads to pancreatic β-cell failure. The decline in β-cell mass secondary to increased apoptosis is of the important pathogenic features of T2DM (48, 49) and ER stress is a key factor contributing to β-cell apoptosis (43). Consistent with that, islets from T2DM patients showed increased levels of CHOP. Specifically, pancreata from obese diabetic showed six times higher expression of perinuclear CHOP as compared to those from obese nondiabetic controls (50). Similarly, db/db mice, an animal model of T2DM, showed existence of ER stress in their islets. In those animals a variety of ER stress marker genes (XBP1, DNAJC3, ATF4, CHOP, BiP) were upregulated in pancreatic islets. In parallel, increased islet expression of DNAJC3, CHOP, BiP proteins in human pancreas sections of T2DM subjects was reported (51). Recent reports have demonstrated that defects in proinsulin/insulin and ER stress markers progressively increase during the transition from normal glucose tolerance to impaired glucose tolerance to eventually T2DM (50). These changes are directly associated with the initial loss of β-cell identity (52). These *in vivo* studies indicate that the progression toward T2DM is characterized by increased expression of ER stress-related genes and increased in β-cell workload (high insulin demand and insulin resistance) that consequently leads to loss of β-cell identity and its dysfunction. Collectively, these findings provide strong evidence that ER stress within pancreatic β-cells may be a crucial contributing factor to β-cell apoptosis in the pathogenesis of polygenic diabetes. Thus, therapeutic intervention aiming at reducing ER stress may alleviate β-cell workload, and consequently delay β-cell failure in T2DM. Accordingly, increasing the UPR capacity of the ER may represent a promising potential therapeutic strategy for preventing the development of polygenic diabetes. One approach that has been reported consists of the use of pharmaceutical compounds with chaperone-like properties such as taurine-conjugated ursodeoxycholic acid derivative (TUDCA) and 4-phenylbutyric acid (PBA) that have the ability the improve the ER folding capacity (53, 54). In humans, administration of PBA has been reported to partially alleviate lipid-induced insulin resistance and β-cell dysfunction. Similarly, in mouse model of T2DM (ob/ob mice), both PBA and TUDCA exhibit ER stress-reducing properties, potentially contributing to restoration of plasma glucose homeostasis and systemic insulin sensitivity (55); indicating that chemical chaperones have potent antidiabetic property through enhancing the adaptive capacity of the ER.

Besides, pancreatic β-cell ER stress is reported to be directly involved in the pathogeny of some forms of monogenic diabetes (11). Monogenic diabetes, which accounts for 1–5% of all diabetes cases, is a spectrum group of inherited disorders caused by mutations in a single gene. Based on the age of appearance, it is clinically divided into (i) maturity-onset diabetes of the young (MODY) and (ii) neonatal or early-onset diabetes mellitus. Currently, more than 40 subtypes of monogenic diabetes have been identified, with the most prevalent being MODY (56). In contrast to the polygenic DM in which environmental factors play a crucial role, the monogenic forms of DM result from mutations or changes in a single gene providing undoubted evidence for the crucial role of genetics in the pathogeny of DM. Interestingly, studies have shown that defects in some MODY genes can cause ER stress in β-cell and subsequently its dysfunction.

MODY-1 is caused by a point mutation of the hepatocyte nuclear factor 4α (HNF4α) gene. HNF4α is known to target and activate Ankyrin Repeat And Sterile Alpha Motif Domain Containing 4B (ANKS4b) in pancreatic β-cells (57). ANKS4B binds to the ER chaperon protein BiP, leading to its overexpression and consequently, enhanced ER stress response. Conversely, suppression of ANKS4B reduced β-cell susceptibility to ER stress-induced apoptosis; indicating that ANKS4b represents a molecular target by which HNF4α regulates ER stress in β-cells (57) and therefore explaining the possible mechanism underlying the loss of HNF4α mediating β-cell dysfunction in MODY-1.

MODY-2 is an autosomal dominant form of monogenic diabetes due to point mutations of the glucokinase (GCK) gene (58). In mammalian, GCK (or hexokinase IV) represents the initiating enzyme of the glycolytic pathway and functions as “glucose sensor” expressed mainly in hepatocytes and pancreatic β-cells. GCK is a key enzyme essential for glucose metabolism that catalyzes the conversion of glucose to glucose-6-phosphate and thus controls GSIS. Thus, reduced GCK activity in β-cell has been reported as the primarily contributor to hyperglycemia in MODY2. Furthermore, mice with a missense mutation in the GCK gene showed defects in β-cell function associated with increased abundance of CHOP expression in their islets, a pro-apoptotic transcription factor involved in the ER stress response (59). By contrast, in Akita mice, a model of ER stress–mediated diabetes, glucokinase activator administration has been shown to improve ER stress–induced apoptosis in pancreatic β-cells by suppressing the expressions of CHOP and Bcl2-associated X protein (Bax) (60); highlighting the role of ER stress in the pathogeny of MODY-2.

MODY-3 is caused by loss-of-function mutations in the gene that encodes hepatocyte nuclear factor 1α (HNF1 α). Interestingly, dysfunction of HNF1A down-regulates XBP1 and BiP expression (61). Moreover, expression of a dominant-negative of HNF1A specifically in pancreatic β-cells induces a MODY-3-like phenotype in mice characterized by increased sensitization of β-cells to ER stress (62).

Neonatal diabetes, as like MODY, is generally caused by single gene mutations and consequently impair β-cell function. YIPF5 gene involved in protein trafficking between the ER and the Golgi organelles is recently reported to play an important role in pancreatic β-cell function. Gain- and loss-of-function studies revealed that patients with homozygous mutations in the YIPF5 gene develop neonatal/early-onset diabetes (63). More importantly, the loss of YIPF5 function-mediated β-cell dysfunction resulted in uncontrolled accumulation of proinsulin in the ER and increased β-cell vulnerability to ER stress-induced apoptosis (63). Additionally, it has been demonstrated that loss-of-function mutations in DNAJC3 (an Hsp40 family member that interacts with PERK) cause early-onset diabetes by increasing sensitization of β-cells to ER stress (64). These findings further highlight the important role of ER stress in the physiopathology of monogenic diabetes.

## 5 Role of ER stress in the functionality of hPSC-derived pancreatic β-cells

**Figure 2:**
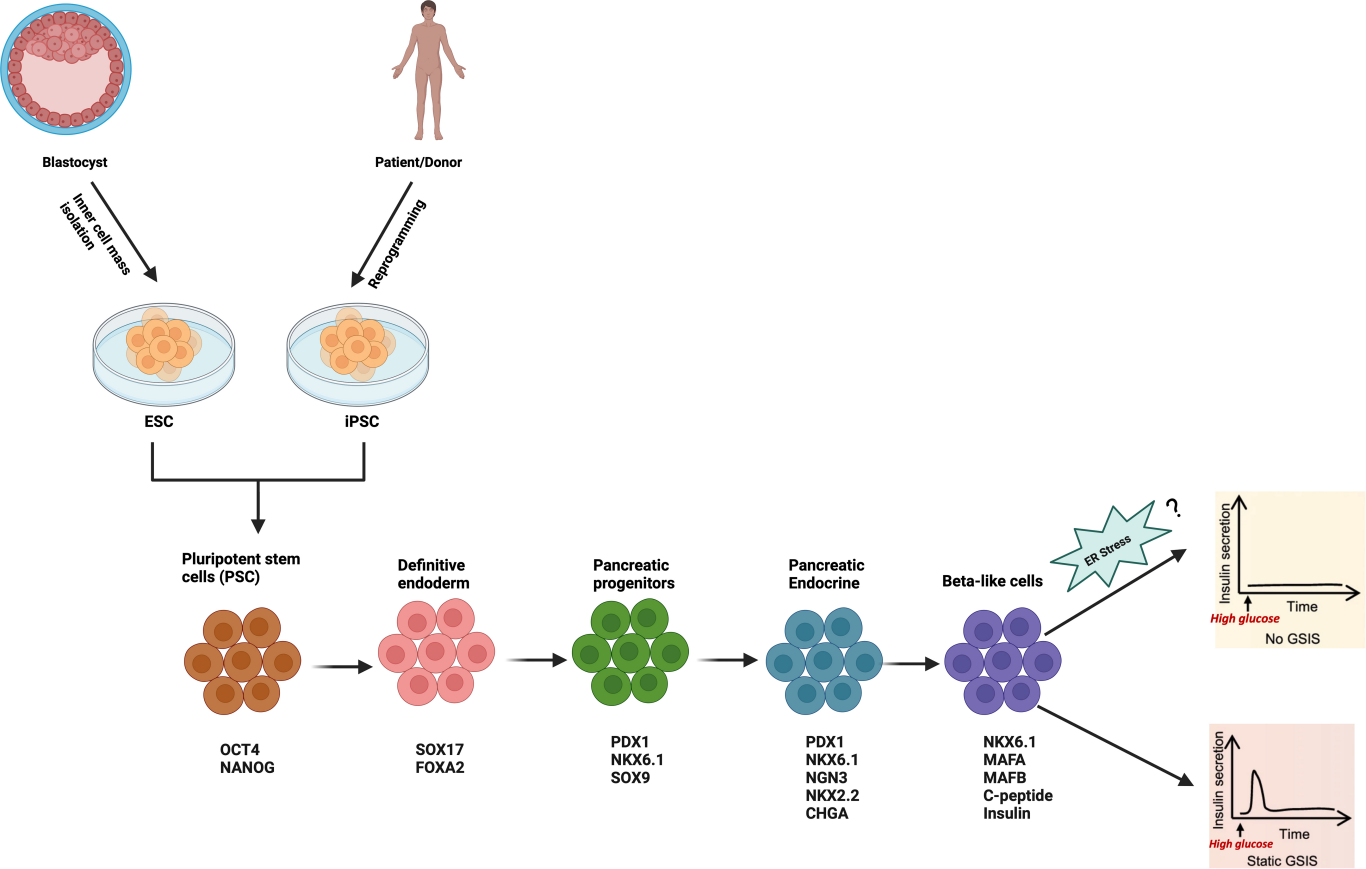
Schematic of the stepwise differentiation protocol for generation of hPSC-derived β-cell. Relevant stage-specific transcription factors and/or cell-surface markers to characterize each stage of the differentiation are also indicated, and they are evaluated at each stage to track the efficiency of differentiation using flow cytometry, immunofluorescence, RT-PCR or Western Blot. Illustration created with Biorender.com.

Human embryonic stem cells (ESC) and induced pluripotent stem cells (iPSC), collectively termed human pluripotent stem cells (hPSC) are unique cells that have the ability to be differentiated virtually into any cell types in the body including pancreatic β-cells due to their infinite self-renewal competency. Generation of transplantable human β-cells from hPSC hold great promise for diabetes therapy (65). Moreover, hPSC-derived β-cells from patients with DM are also critical for a better understanding of the disease and its progression, particularly in diabetes related mutations such as the inherited monogenic diabetes (66). To reach that goal, massive efforts have been undertaken over the last two decades to efficiently differentiate hPSCs into insulin-expressing β-cells using multi-stage directed differentiation protocols imitating all stages of pancreas embryogenesis from definitive endoderm (DE) to maturing β-cells. These directed differentiation protocols occur by first inducing DE, followed by generation of primitive gut tube (PGT), posterior foregut (PF), pancreatic progenitor (PP), endocrine precursors (EP) and finally β-cells (**Figure 2**). Specific transcription factors and/or cell-surface markers are used to identify each stage of the differentiation. However, the limited functional maturation (impaired GSIS) of hPSC-derived insulin-expressing β-cells emanating from those protocols hampers the current strategies of cell replacement therapy for diabetes. Multiple mechanisms are involved in the impaired GSIS. ER and mitochondria are two pivotal organelles involved in the function and survival of β-cells (11, 67). *In vivo* and *in vitro* studies have highlighted distinct or combined role of ER stress and mitochondria dysfunction in β-cell functionality (11, 68).

As highlighted in previous sections, transcriptome studies on both human and mouse islets and β-cells, as well as polygenic and monogenic forms of DM have provided compelling evidence on the importance of ER stress and UPR in β-cell dysfunction and diabetes. However, the exact role of ER stress on the functionality of hPSC-derived β-cells is not well characterized. Current hPSC-derived β-cell differentiation methods involves multi-step stages lasting several weeks, during which the cells are exposed to a complex cocktail of small molecules/growth factors/cytokines along with alternance of low and high glucose concentration in basal media (e.g. Melton 3D protocol (6) S5 media contains 20mM of glucose as compared to 2.5mM in S1 and S3 media) to either activate or repress key stage-specific transcription factors for lineage specification signals to guide β-cell differentiation and promote maturity. However, considering the high vulnerability of hPSC to ER stress (69), the daily exposure to this complex cocktail along with chronic high glucose (glucotoxicity) may induce metabolic stress that subsequently lead to a perturbation of the ER homeostasis and may negatively affect the fate and function of the cells. To our knowledge, no study has investigated the potential influence of the small molecules and media used across all stages of the differentiation on ER stress. Considering the importance of UPR for normal β-cell function, it is possible that increased ER stress or defects in ER signaling pathways could represent the main contributing factor to the immature phenotypes observed in hPSC-derived pancreatic β-cells *in vitro* (**Figure 2**). More recently, imeglimin, an antidiabetic agent, was reported to improve hPSC-derived pseudo-islet’s functional maturation by modulating the ER homeostasis pathway (70). Specifically, it increased the expression of ER-related molecules such as CHOP, ATF3, and restored the global protein synthesis in β-cells under ER stress. Consistent with that, loss-of-function of Solute Carrier Family 30 (Zinc Transporter), Member 8 (SLC30A8 or ZnT8), a zinc transporter reported to be mainly expressed in pancreatic β-cells and negatively associated with β-cell function, has been demonstrated to accelerate functional maturation in CRISPR)/Cas9-mediated SLC30A8 knock out stem cell-derived β-cells (71). Mechanistically, it improves GSIS by alleviating ER stress evidenced by down-regulation of IRE1α, XBP1, and sXBP1, thus providing a proof of concept that the functionality of hPSC-derived β-cells can be enhanced by targeting ER stress via either gene editing (e.g. CRISPR)/Cas9 technology) or pharmacological agents (e.g. imeglimin). These findings demonstrate convincingly that targeting ER stress could emerge as a promising strategy to further enhance the differentiation protocols for generation of mature-like and functional hPSC-derived β-cells to faithfully phenocopy the response of bona fide human β-cells, potentially for diabetes therapy or drug screening. However, due to the off-target cutting effects at other sites in the genome of current available gene editing tools such as CRISPR/Cas9 and Zinc Finger Nuclease (ZFN) and Transcription Activator-Like Effector Nuclease (TALEN) (72), there are caveats to their use that must be taken into consideration when studying the role of ER stress in hPSC- derived β-cells.

## 6 Conclusion

Multiple known and unknown mechanisms are involved in β-cell dysfunction associated with impaired insulin secretion and diabetes mellitus. Mounting evidence implicate ER stress as a contributing factor to insulin secretory defects in diabetic patients. To restore ER homeostasis, similarly to many cells in the body, β-cells activate the ER stress response (namely UPR) in their protein-folding machinery, underscoring the importance of this process for normal β-cell function. As β-cells are intrinsically susceptible and constantly exposed to ER stress owing to an intense trafficking of proinsulin to the ER due to high demand for insulin production and secretion in response to dietary nutrient stimulation, chronic supra-physiological ER stress in β-cells alters the UPR signaling that eventually leads to β-cell demise and diabetes. In this article we explore and summarize recent advances in elucidating the role of ER stress and its associated signaling mechanisms on pancreatic β-cell function and diabetes and how ER stress pathways could be targeted *in vitro* during direct differentiation protocols for generation of functional hPSC-derived pancreatic β-cells for cell replacement therapy for diabetes. More importantly, due to limited access to human pancreas samples, patient-specific hPSC-derived β-cells, also known as autologous hPSC-derived β-cells, can be a useful tool *in vitro* to gain a better understanding of the relationship between ER stress and diabetes. Additionally, modulation of ER stress with chemical chaperones (e.g. glycerol, trehalose, TUDCA, 4-PBA) during direct differentiation protocol may improve the functionality of hPSC-derived β-cells that recapitulate all phenotypic characteristics of the human pancreatic β-cell.

## Author contributions

AD: Writing – original draft, Writing – review & editing. AA: Writing – review & editing. RM: Writing – review & editing. HA: Writing – review & editing.
